# Aberrant expression of long noncoding RNAs in chronic thromboembolic pulmonary hypertension

**DOI:** 10.3892/mmr.2014.3102

**Published:** 2014-12-17

**Authors:** SONG GU, GUANGHUI LI, XITAO ZHANG, JUN YAN, JIE GAO, XIANGGUANG AN, YAN LIU, PIXIONG SU

**Affiliations:** Department of Cardiac Surgery, Beijing Chaoyang Hospital, Capital Medical University, Beijing 100020, P.R. China

**Keywords:** chronic thromboembolic pulmonary hypertension, mRNA, long noncoding RNA, microarray, gene ontology

## Abstract

Chronic thromboembolic pulmonary hypertension (CTEPH) is one of the primary causes of severe pulmonary hypertension. In order to identify long noncoding RNAs (lncRNAs) that may be involved in the development of CTEPH, comprehensive lncRNA and messenger RNA (mRNA) profiling of endothelial tissues from the pulmonary arteries of CTEPH patients was conducted with microarray analysis. Differential expression of 185 lncRNAs was observed in the CTEPH tissues compared with healthy control tissues. Further analysis identified 464 regulated enhancer-like lncRNAs and overlapping, antisense or nearby mRNA pairs. Coexpression networks were subsequently constructed and investigated. The expression levels of the lncRNAs, NR_036693, NR_027783, NR_033766 and NR_001284, were significantly altered. Gene ontology and pathway analysis demonstrated the potential role of lncRNAs in the regulation of central process, including inflammatory response, response to endogenous stimulus and antigen processing and presentation. The use of bioinformatics may help to uncover and analyze large quantities of data identified by microarray analyses, through rigorous experimental planning, statistical analysis and the collection of more comprehensive data regarding CTEPH. The results of the present study provided evidence which may be helpful in future studies on the diagnosis and management of CTEPH.

## Introduction

Pulmonary endarterectomy (PEA) can prevent mortality due to right ventricle failure (1), which occurs in chronic thromboembolic pulmonary hypertension (CTEPH), a type-4 pulmonary hypertension (2). A number of gene expression studies conducted in postmortem lung tissue samples from patients with CTEPH have indicated that aberrant processing of the messenger RNA (mRNA) transcriptome in CTEPH may provide a mechanistic convergence between the diverse genetic heritability of this disease and the disruption of fundamental signaling pathways, resulting in the common CTEPH phenotype. Studies conducted in pulmonary endothelial cells have identified differentially expressed genes, which may be involved in the pathogenesis of CTEPH (3,4). However, gene expression regulation is a complex process, which involves an interplay between DNA sequence variation, chromatin and epigenetic modifications, protein transcription factors and regulatory noncoding RNAs.

A previous study by our group examining the role of the transcriptome in pulmonary artery endomembrane samples demonstrated that the abnormal expression of mRNA transcripts may represent a point of convergence in the otherwise heterogeneous genomics, underlying the development of CTEPH (5). However, the regulatory RNAs inducing the aberrant mRNA expression levels observed in CTEPH have not been concurrently assessed. To the best of our knowledge, only a single study conducted in the tissues of CTEPH patients has demonstrated that miRNA-759 may influence the susceptibility to the development of CTEPH (6). Long noncoding RNAs (lncRNAs), a novel class of regulatory RNAs, have been shown to be involved in certain fundamental events in gene regulation. However, the role of these molecules in the pathogenesis of CTEPH remains unclear (7).

lncRNAs are noncoding RNA molecules that are longer than 200 nucleotides. lncRNAs were originally considered to be ‘transcriptional noise’; however, their involvement in important mechanisms controlling the gene expression regulation has been demonstrated. These mechanisms include targeting transcription factors, initiating chromatin remodeling, directing methylation complexes and blocking proximate transcription (8). Aberrant regulation of lncRNAs has been shown to be associated with a number of diseases, including certain forms of cancer (9,10). Numerous lncRNAs have been identified through large-scale analyses of full-length cDNA sequences in humans, mice and flies. lncRNA molecules have been shown to play an important role in the control of imprinting, cell differentiation, immune response, pathogenesis of various human diseases, tumorigenesis and other biological processes (11–15). However, the expression and biological function of lncRNAs in CTEPH remains to be elucidated.

The aim of the present study was to determine whether the dysregulation of the lncRNA expression is involved in the molecular pathogenesis of CTEPH. The lncRNAs expression profiles of five CTEPH patients were compared with healthy control individuals (normal tissues). In addition, an assessment of the transcriptional differences in all known protein-coding mRNAs was conducted.

## Materials and methods

### Patient samples

In total, five patients diagnosed with CTEPH (male, 2; female, 3; mean age, 38.2 years; age range, 17–52 years) who had been referred to Beijing Chaoyang Hospital (Capital Medical University, Beijing, China) were recruited to the study. The study was approved by the relevant ethics committee of Beijing Chaoyang University. All the patients provided informed written consent prior to participation in the study. Pulmonary angiography and right heart catheterization were used in the diagnosis of CTEPH and determination of cardiopulmonary hemodynamics (16). Mean pulmonary artery pressure >25 mmHg at rest or >30 mmHg during exercise was considered to indicate the presence of pulmonary hypertension. Pulmonary vascular resistance (PVR) and the six-minute walk test (6-MWT) were hemodynamic variables applied to assess the cardiopulmonary function and prognosis of the CTEPH patients. At inclusion, all the patients received oral anticoagulants for a minimum of 6 months and underwent PEA in accordance with the guidelines of the Beijing Chao-Yang Hospital (Beijing, China). In addition, healthy control samples were obtained from five lung transplant donors. The control subjects and patients were matched according to age and gender. Written informed consent was obtained from the healthy controls or their families.

### RNA extraction

To prepare the samples for microarray profiling, total RNA was isolated from the CTEPH patient and normal tissue samples using TRIzol™ reagent (Invitrogen Life Technologies, Inc., Burlington, ON, Canada) and purified using an RNeasy Mini kit (Qiagen, Hilden, German), including a DNase digestion treatment. RNA concentrations were determined by measuring the sample absorbance at 260 nm with a NanoDrop 2000 spectrophotometer (Thermo Fisher Scientific, Inc., Waltham, MA, USA). A260/A280 ratio values of 1.8–2.1 were set as the quality control standard.

### Microarray profiling

cDNA was generated via reverse transcription of RNA obtained from pulmonary artery endothelium samples using a reverse transcription kit (miScript II RT kit; Qiagen). cDNA obtained from pulmonary artery endothelium samples of the CTEPH patients or normal controls was hybridized to GeneChip^®^ Human Gene 2.0 ST arrays (Affymetrix, Inc., Santa Clara, CA, USA), according to the manufacturer’s instructions. Affymetrix Expression Console™ software (version 1.2.1; Affymetrix, Inc.) was used for microarray analysis. The raw data (CEL files) were normalized at the transcript level using the robust multiarray average method (17), followed by median summarization of the transcript expression levels. Subsequently, gene-level data were filtered to include only the probe sets derived from the ‘core’ metaprobe list, representing the reference sequence (RefSeq) genes.

### Significant differential gene analysis

The random variance model (RVM) t-test was used to filter differentially expressed genes in the control and CTEPH groups. This test was selected since it can effectively raise the degrees of freedom when investigating small samples. Following significance analysis of microarrays and false discovery rate (FDR) analysis, the differentially expressed genes were identified according to the predetermined P-value threshold using BRB-ArrayTools (version 4.3.0 Beta 1; National Cancer Institute, Bethesda, MD, USA). P<0.05 was considered to indicate a statistically significant difference (18–20). The differentially expressed genes were subjected to unsupervised hierarchical clustering (Cluster 3.0; Stanford University, Stanford, CA, USA) and TreeView version 3.0 analysis (Stanford University).

### Coexpression network

Gene coexpression networks were constructed based on the normalized signal intensity of the specific expression of genes, in order to identify the interactions among genes (21). For each pair of genes, the Pearson correlation coefficient was calculated in order to identify pairs with a significant correlation, thus enabling construction of the network (22).

When conducting a network analysis, the simplest and most important measure of gene centrality within a network is degree centrality. Degree centrality is defined as the number of links a particular node has to other nodes in the network (23). Furthermore, k-cores were introduced using graph theory in order to simplify the graph topology analysis and investigate various network properties. A k-core of a network consists of a subnetwork where all the nodes are connected to at least k other genes. A k-core of a protein-protein interaction network usually contains cohesive groups of proteins with similar functions (23,24).

Network structure analysis aims to locate core regulatory factors (genes). Within a network, core regulatory factors connect the majority of nearby genes and have the highest degree centralities. When evaluating different networks, core regulatory factors are determined by the degree differences between the CTEPH and normal tissue samples (25), since they show the highest degree differences. The network was constructed using Cytoscape software version 2.8.3 (Cytoscape Consortium, San Diego, CA, USA).

### Gene ontology (GO) analysis

Based on the gene ontology database (http://www.geneontology.org/; accessed on January 15, 2014), the significance level of GO terms for the CTEPH-associated differentially expressed genes was analyzed by two-side Fisher’s exact test and χ^2^ test using the Database for Annotation, Visualization and Integrated Discovery (DAVID) software version 6.7 (http://david.abcc.ncifcrf.gov/home.jsp; accessed on January 11, 2014; National Institute of Allergy and Infectious Diseases, National Institutes of Health, Bethesda, MD, USA) (26). Differentially expressed genes were analyzed independently according to whether they were upregulated or downregulated. P-values were calculated for the differentially expressed genes in all GO categories. P<0.01 and FDR<0.01 were considered to indicate statistically significant results.

### Pathway analysis

Based on the Kyoto Encyclopedia of Genes and Genomes database (http://www.genome.jp/kegg/; accessed on January 12, 2014; Kanehisa Laboratories, Kyoto, Japan), the significance levels of CTEPH-associated differentially expressed gene pathways were analyzed using Pathway-Express version 1.0 (Intelligent Systems and Bioinformatics Laboratory, Detroit, MI, USA) (27,28). The occurrence of significant differences from the expected values was assessed using a two-sided binomial distribution. The number of differentially expressed genes corresponding to each pathway category was counted and compared with the number of genes expected for each pathway category. All the signaling pathways were analyzed using γ P<0.05, provided by the impact analysis, as the threshold indicating a statistically significant difference.

## Results

### Overview of lncRNA profiles

Based on the lncRNAs expression profiles, a number of differentially expressed lncRNAs were identified between the CTEPH and healthy control samples. The expression profiles of lncRNAs in the paired samples were determined by calculating the log fold change of CTEPH/control samples. Due to the limited sample size, FDR and P-values were calculated from normalized expression levels. Hundreds of differentially expressed human lncRNAs were identified using the RefSeq (www.ncbi.nlm.nih.gov/refseq), Ensembl (www.ensembl.org), lncRNAdb (www.lncrnadb.org), Broad Institute, Human Body Map lincRNAs (www.broadinstitute.org/genome_bio/human_lincrnas/) and transcripts of uncertain coding potential catalog (http://www.broadinstitute.org/genome_bio/human_lincrnas/?q=TUCP_transcripts_catalog) databases in the CTEPH patients and healthy controls.

**Figure 1:**
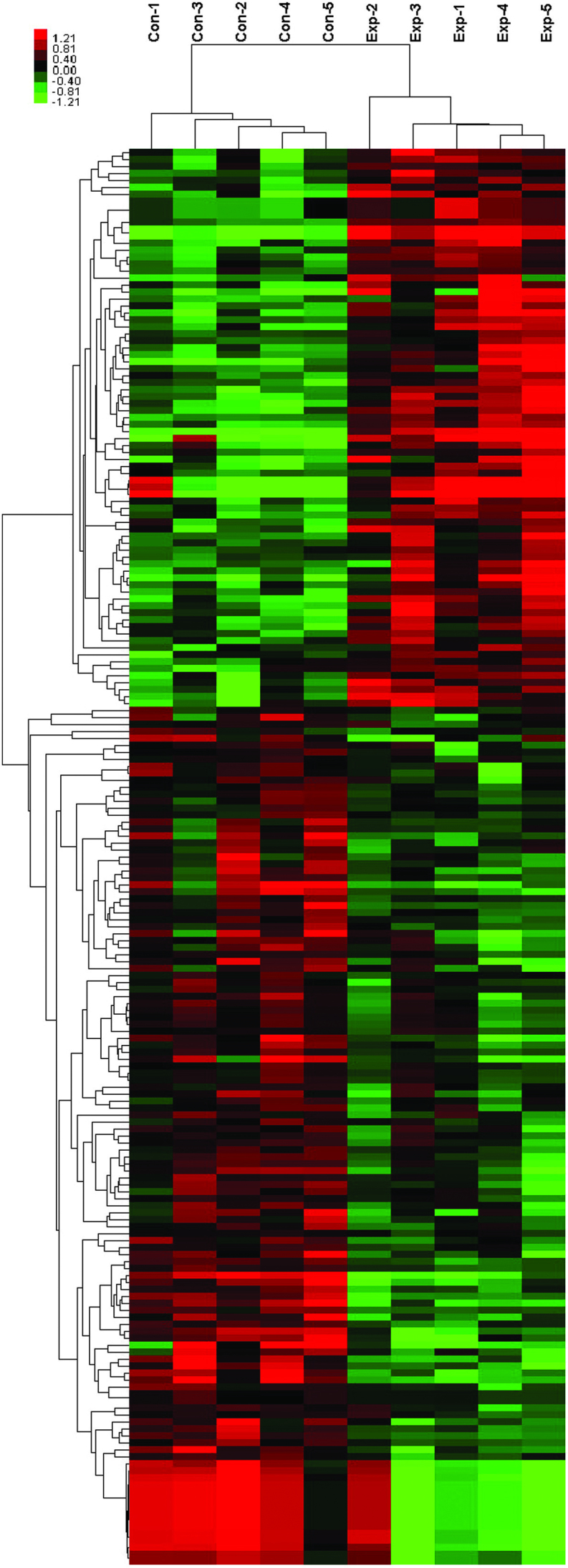
Unsupervised classification of samples from chronic thromboembolic pulmonary hypertension patients and healthy controls, based on long noncoding RNA expression profiling. The data are depicted as a data matrix with rows representing the probes and columns representing the samples. The expression levels are presented according to the color scale shown at the top. Red and green indicate the expression levels above and below the median, respectively. The magnitude of deviation from the median is represented by the color saturation. Con, control group; Exp, experimental group.

Using the microarray data, the expression levels of lncRNAs in the five CTEPH tissue samples were compared with the matched normal tissue samples. In total, 185 lncRNAs were identified in which the expression levels were found to be significantly different between the two groups. The characteristics of the five CTEPH patients and five normal controls are shown in Table I. The procedure used to obtain a sequence may vary depending on the database used for lncRNA classification. While tens of thousands lncRNAs were investigated in normal and diseased tissues, only a few hundreds lncRNAs were found to be significantly upregulated or downregulated. Thus, the upregulation or downregulation of lncRNAs was used to distinguish the CTEPH patient tissues from healthy tissues (Fig. 1). Compared with normal tissues, NR_033766 was the most evidently downregulated lncRNA, whereas TCONS_l2_00010131-XLOC_l2_005462 was upregulated to the greatest degree (Table II). Therefore, downregulated lncRNAs were shown to be more prevalent compared with upregulated lncRNAs in the CTEPH group.

### Overview of mRNA profiles

In total, ≤30,654 coding transcripts were detected in the ten samples that were examined. Using the RVM t-test method, 880 genes were found to be upregulated and 734 genes were found to be downregulated in the CTEPH samples, compared with the healthy controls. The results supported the hypothesis that CTEPH is a metabolic disease as the mRNA expression level of oxidized low-density lipoprotein receptor 1 showed the greatest upregulation, while the mRNA expression level of chordin-like 1 showed the greatest downregulation. Therefore, upregulation of mRNA expression levels was more prevalent compared with downregulation in the CTEPH group.

### Analysis of nearby lncRNAs and mRNAs

Previous studies have used chromatin-state maps to identify 3,019 lncRNAs with a clear evolutionary conservation, which are associated with distinct and diverse biological processes (such as cell proliferation), RNA binding complexes, immune surveillance, embryonic stem cell pluripotency, neuronal processes, morphogenesis, gametogenesis and muscle development (29,30). Among the 185 differentially expressed lncRNAs identified in the present study, 74 lncRNAs were shown to have differentially expressed mRNAs that were overlapping, antisense or nearby. Further analysis resulted in the identification of nine pairs of differentially expressed lncRNAs overlapping with mRNAs, nine pairs of lncRNAs and antisense mRNAs, 340 lncRNAs located upstream of mRNAs (distance, <300 kb) and 106 lncRNAs located downstream of mRNAs (distance, <300 kb) in each comparison between CTEPH and normal control tissues. Among the 464 lncRNA-mRNA pairs, the expression regulation of 442 lncRNAs and nearby coding genes was in the same direction (up or down), whereas the expression of 22 pairs was regulated in opposite directions.

### Construction of the coding-noncoding gene coexpression network

**Figure 2:**
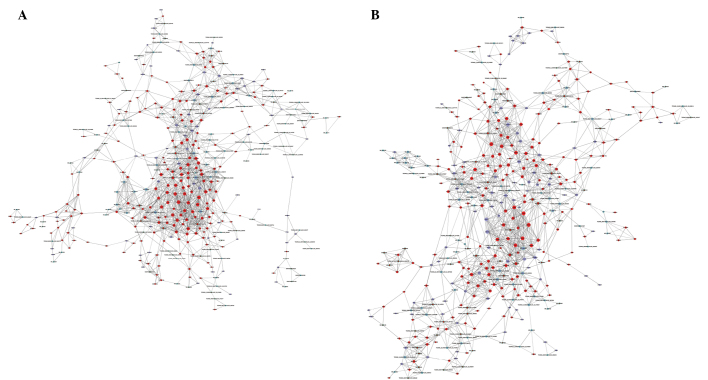
Coding-noncoding gene coexpression network of (A) chronic thromboembolic pulmonary hypertension and (B) normal control groups. Blue represents downregulation and red represents upregulation. Circle nodes represent messenger RNA (mRNA), while rim nodes represent long noncoding RNA (lncRNA). Solid lines represent a positive regulatory association and dashed lines represent a negative regulatory association between lncRNA and mRNA.

The correlation analysis among differentially expressed lncRNAs and mRNAs was used to construct a coding-noncoding (CNC) gene coexpression network. LncRNAs and mRNAs having Pearson correlation coefficients ≥0.97 were selected and the network was constructed using the Cytoscape software. Within this coexpression network, the CTEPH-CNC network node consisted of 129 lncRNAs and 275 mRNAs, whereas the normal control-CNC network node consisted of 134 lncRNAs and 294 mRNAs (Fig. 2). A total of 832 network nodes in the two networks formed 3,239 coexpression pairs of lncRNAs and mRNAs, with positively correlated expression in the majority of pairs. Investigation of the CNC network indicated that one mRNA may correlate with numerous lncRNAs and vice versa.

In order to identify the most significant RNA molecules in CTEPH, the degree of certain RNAs (k-core) in each network was normalized and the difference in connectivity (diffK), representing the differences between the two networks, was calculated. NR_036693 and NR_027783 were found to be the most differentially expressed lncRNAs, whilst the expression of the arginine vasopressin receptor 1A gene showed the greatest significant difference in the genes examined. The CNC network presented here, may implicate the inter-regulation of lncRNAs and mRNAs in the development of CTEPH.

### Coexpressed coding gene function analysis

**Figure 3:**
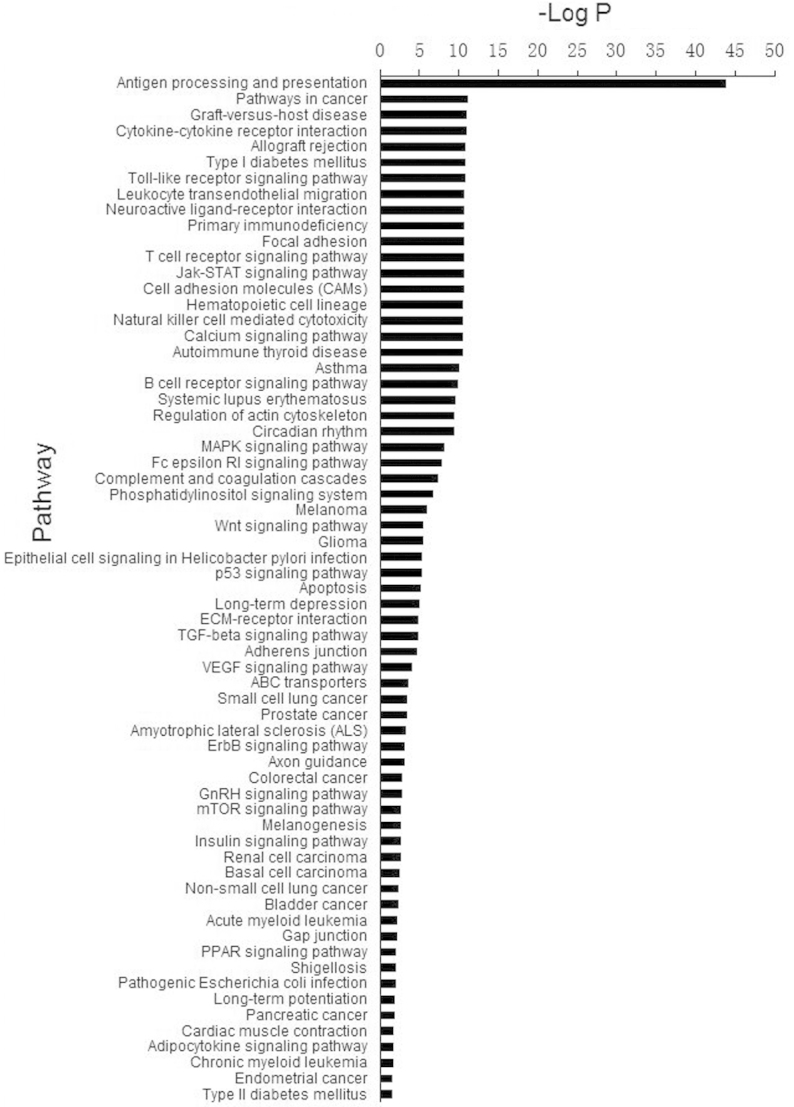
Histogram of signaling pathways that were found to be significantly different between the chronic thromboembolic pulmonary hypertension and normal control groups. −log P, negative logarithm of P-value (larger −log P values indicate smaller P-values).

An important part of research into lncRNAs involves inferring the possible functions of nearby protein-coding genes (29,31). In the current study, the differentially expressed mRNAs of the two CNC networks were combined. Subsequently, the DAVID functional annotation chart (32,33) and pathway analysis results were used to perform functional enrichment analysis of the differentially regulated protein-coding gene and lncRNA pairs. The annotation terms showing the greatest significant differences (with the lowest P-values) were found to be immune response, inflammatory response, defense response and response to wounding (Table III). In addition, the most important signaling pathways relevant to CTEPH were found to be antigen processing and presentation, cytokine-cytokine receptor interaction and leukocyte transendothelial migration (Fig. 3). Therefore, lncRNAs are hypothesized to modulate the host response through their effect on nearby protein-coding genes.

## Discussion

To the best of our knowledge, no previous studies have investigated the lncRNA expression profiles in CTEPH or the association of lncRNA expression with clinical characteristics and outcomes in CTEPH patients. CTEPH is a polygenic disorder, resulting from genetic alterations. The description of thousands of genomic sequences, along with technological developments enabling the identification of gene expression profiles on a large scale, have improved the understanding of the pathogenesis of a number of diseases, including cancer (34). These advances may also facilitate the development of novel therapeutic targets, as well as diagnostic and prognostic markers. Thus, a concerted effort to genetically characterize CTEPH may provide an improved understanding of the pathogenesis and development of the disease, as well as help identify novel personalized treatments. A number of studies have demonstrated that the expression levels of lncRNAs are dysregulated in numerous human diseases, including lung cancer and hepatocellular carcinoma (35–37,38). In addition, lncRNAs, such as HOTAIR, are involved in the development and progression of tumors, such as breast cancer (39).

In the current study, differentially expressed lncRNAs and nearby coding gene pairs were described. The silencing or reduced expression of particular lncRNAs has been previously demonstrated to result in a concomitant reduction in the expression of nearby protein-coding genes, including numerous proteins which are known to govern the regulation of cellular differentiation. In addition, the lncRNAs and nearby coding genes may share upstream regulation or local transcriptional effects (29,31,40–42). Certain lncRNAs have been reported to increase gene expression. For instance, Evf-2 ncRNA forms a complex with the homeodomain-containing protein Dlx2, which leads to transcriptional enhancement (43). In addition, heat-shock RNA-1 ncRNA binding to heat-shock transcription factor 1 has been found to result in the induction of heat-shock proteins (44). Furthermore, an isoform of the ncRNA steroid receptor RNA activator is known to be associated with steroid receptor responsiveness (45). Finally, Ørom *et al* (46) recently identified that noncoding RNA-activators 1–7 can enhance the expression of nearby genes. Thus, analyzing the genes nearby to lncRNAs may assist in understanding the involvement of lncRNAs in CTEPH. The majority of lncRNAs have a distinct spatial and temporal specificity in the process of organismal differentiation and development (47). A previous study, which investigated 1,300 lncRNAs in mice, demonstrated that in particular areas of the brain, there are different expression patterns of lncRNAs (48). These lncRNA expression signatures have been detected in prostate carcinoma and hepatic tumors (49). Thus, differential expression patterns of lncRNAs may be present in the pulmonary artery tissues of CTEPH patients, and lncRNAs that are differentially expressed may result in alterations in cellular function, which may be associated with the pathogenesis of CTEPH. In the present study, 464 pairs of differentially expressed enhancer-like lncRNAs and mRNAs, of which 95.3% (442/464) were regulated in the same direction (up or down). Therefore, the present study hypothesized that a number of these lncRNAs enhance the activation of nearby genes.

Molecular networks are useful in the investigation of biological processes and can be constructed using results obtained from co-immunoprecipitation experiments (50) or from algorithmic predictions based on gene function correlation and expression profiles (51). Network models based on algorithmic predictions from high throughput gene expression tests may be used to construct images of the networks regulating gene expression and metabolic pathways in the groups analyzed. The networks intrinsic to the CTEPH phenotype are hypothesized to be involved in the normal functioning of the pulmonary artery endothelium. Based on the information obtained regarding the expression of lncRNAs and mRNAs, Pearson correlation coefficients were calculated. Pairs found to have a significant correlation were selected and used to construct a network. These results demonstrated an association between lncRNAs and mRNAs, indicating that lncRNAs may regulate specific mRNAs, or vice versa. mRNAs are likely to be directly involved in the pathogenesis of CTEPH, while lncRNAs function through the epigenetic modification of mRNAs.

Based on previous studies and the results of the computer analysis conducted in the present study, four lncRNAs with the largest diffK were further investigated. lncRNA NR_036693 is a 5,255 bp transcript variant 6 of *Homo sapiens* C-type lectin domain family 2, member D. This gene encodes a member of the natural killer cell receptor C-type lectin family. The natural killer cell has been found to be involved in vascular remodeling, which may lead to pulmonary arterial hypertension (52,53). NR_027783 is a 1,199 bp transcript variant 2 from *Homo sapiens* spermidine/spermine N1-acetyltransferase 1 (SAT1). The protein encoded by this gene is a member of the acetyltransferase family and a rate-limiting enzyme in polyamine metabolism. Numerous studies have demonstrated that the polyamine regulatory pathway is a pharmacological target in pulmonary arterial hypertension (54,55). NR_033766 is a 6,384 bp transcript variant 7 from *Homo sapiens* forkhead box P2 (FOXP2). The FOXP2 gene is involved in the normal development of the areas of the brain controlling speech and language during embryogenesis. In addition, FOXP2 may be associated with a number of biological pathways and cascades, which also influence the development of language. Mutations in this gene result in speech-language disorder 1 (SPCH1), also termed as autosomal dominant speech and language disorder with orofacial dyspraxia. NR_001284 is a 2,783 bp pseudogene, *Homo sapiens* tenascin XA, the biological function of which remains unclear.

Identification of the putative functions of genes associated with lncRNAs may improve the understanding of the functional role of these molecules (30,32). Peng *et al* (56) performed a functional enrichment analysis on protein-coding genes nearby to differentially expressed lncRNAs in SARS-CoV infected mice. The authors identified that the most significant functional group consisted of annotation terms associated with gene expression, including transcription regulation, nuclear and DNA-binding transcription factor activity, as well as the regulation of RNA metabolism. In the present study, GO functional enrichment analysis of differentially expressed mRNAs with their coexpressed differentially expressed lncRNA partners, demonstrated that these genes were functionally associated with immune response, inflammatory response, response of wounding and response to endogenous stimulus. Furthermore, pathway analysis revealed that antigen processing and presentation, cytokine-cytokine receptor interaction and leukocyte transendothelial migration may be involved in the development of CTEPH. Although the function of lncRNAs in CTEPH still requires further investigation, the present study hypothesized that the formation of CTEPH may be caused by certain lncRNAs.

To the best of our knowledge, this is the first study describing the expression profiles of human lncRNAs in CTEPH by microarray. The expression levels of a number of lncRNAs were found to be aberrant in tissue samples from CTEPH patients, compared with the healthy control tissues. These deregulated lncRNAs may function as activators or suppressors of genes involved in the development and progression of the disease. Further investigation is required to determine whether these lncRNAs may serve as novel therapeutic targets and diagnostic biomarkers in CTEPH.

## Figures and Tables

**Table I:** Clinical characteristics of study participants.

Group	n	Age, years (mean ± SD)	Gender, n (M/F)	Median mPAP (range), mmHg	PVR, dyn × sec × cm^−5^	6WMT, m
Healthy control	5	35.3±10.6	2/3	-	-	-
CTEPH patients	5	38.2±14.7	2/3	55 (33–78)	1,075.4	454.6667

SD, standard deviation; M, male; F, female; mPAP, mean pulmonary artery pressure; PVR, pulmonary vascular resistance; 6MWT, six-minute walk test; CTEPH, chronic thromboembolic pulmonary hypertension.

**Table II:** Collection of the top ten deregulated lncRNAs detected using microarray analysis in ten CTEPH and control samples.

Downregulated in CTEPH tissues			Upregulated in CTEPH tissues		
	
lncRNA	P-value	Fold change^a^	lncRNA	P-value	Fold change^a^
NR_003679	5.70×10^−6^	0.45	TCONS_l2_00010131-XLOC_l2_005462	3.00×10^−5^	5.89
NR_033766	2.14×10^−5^	0.16	TCONS_l2_00011539-XLOC_l2_005705	3.00×10^−5^	5.89
TCONS_l2_00004769-XLOC_l2_002469	3.52×10^−5^	0.41	NR_026544	1.16×10^−4^	2.61
TCONS_00023959-XLOC_011280	4.21×10^−5^	0.49	TCONS_00009277-XLOC_004803	1.18×10^−4^	1.82
TCONS_00023957-XLOC_011280	4.96×10^−5^	0.52	TCONS_l2_00016084-XLOC_l2_008434	1.24×10^−4^	2.02
NR_026799	6.78×10^−5^	0.39	NR_028406	2.36×10^−4^	2.17
TCONS_l2_00020176-XLOC_l2_010319	9.89×10^−5^	0.32	TCONS_00000337-XLOC_000468	4.50×10^−4^	2.42
NR_026985	1.29×10^−4^	0.46	NR_002433	5.00×10^−4^	2.46
TCONS_00000192-XLOC_000173	1.42×10^−4^	0.28	TCONS_00028854-XLOC_013966	5.18×10^−4^	1.89
NR_026597	1.56×10^−4^	0.45	NR_033652	5.66×10^−4^	1.91

a Fold change vs. healthy control tissues.

lncRNA, long noncoding RNA; CTEPH, chronic thromboembolic pulmonary hypertension.

**Table III:** GO analysis.

GO ID	Term	Regulation	P-value	FDR
GO:0006955	Immune response	Up	4.21×10^−59^	7.32×10^−56^
GO:0006952	Defense response	Up	1.24×10^−49^	2.16×10^−46^
GO:0006954	Inflammatory response	Up	5.61×10^−44^	9.77×10^−41^
GO:0009611	Response to wounding	Up	2.88×10^−43^	5.01×10^−40^
GO:0002684	Positive regulation of immune system process	Up	7.37×10^−39^	1.28×10^−35^
GO:0001775	Cell activation	Up	3.95×10^−29^	6.88×10^−26^
GO:0002696	Positive regulation of leukocyte activation	Up	1.01×10^−27^	1.76×10^−24^
GO:0050867	Positive regulation of cell activation	Up	4.41×10^−27^	7.68×10^−24^
GO:0045321	Leukocyte activation	Up	1.56×10^−26^	2.72×10^−23^
GO:0050865	Regulation of cell activation	Up	9.23×10^−26^	1.61×10^−22^
GO:0042110	T cell activation	Up	2.35×10^−25^	4.10×10^−22^
GO:0002694	Regulation of leukocyte activation	Up	2.58×10^−25^	4.49×10^−22^
GO:0046649	Lymphocyte activation	Up	6.86×10^−24^	1.19×10^−20^
GO:0051249	Regulation of lymphocyte activation	Up	5.15×10^−22^	8.98×10^−19^
GO:0051251	Positive regulation of lymphocyte activation	Up	5.97×10^−22^	1.04×10^−18^
GO:0048584	Positive regulation of response to stimulus	Up	1.77×10^−21^	3.09×10^−18^
GO:0042330	Taxis	Up	4.76×10^−21^	8.30×10^−18^
GO:0006935	Chemotaxis	Up	4.76×10^−21^	8.30×10^−18^
GO:0050870	Positive regulation of T cell activation	Up	4.71×10^−19^	8.21×10^−16^
GO:0050863	Regulation of T cell activation	Up	1.18×10^−18^	2.06×10^−15^
GO:0042981	Regulation of apoptosis	Up	5.68×10^−18^	9.89×10^−15^
GO:0043067	Regulation of programmed cell death	Up	8.67×10^−18^	1.51×10^−14^
GO:0010941	Regulation of cell death	Up	1.01×10^−17^	1.77×10^−14^
GO:0001817	Regulation of cytokine production	Up	2.11×10^−17^	3.67×10^−14^
GO:0050778	Positive regulation of immune response	Up	1.73×10^−16^	3.89×10^−13^
GO:0007626	Locomotory behavior	Up	1.26×10^−15^	2.13×10^−12^
GO:0006928	Cell motion	Up	2.59×10^−15^	4.45×10^−12^
GO:0051240	Positive regulation of multicellular organismal process	Up	4.47×10^−15^	7.74×10^−12^
GO:0030098	Lymphocyte differentiation	Up	4.87×10^−15^	8.50×10^−12^
GO:0030217	T cell differentiation	Up	5.86×10^−15^	1.02×10^−11^
GO:0007166	Cell surface receptor linked signal transduction	Up	7.85×10^−15^	1.37×10^−11^
GO:0051094	Positive regulation of developmental process	Up	1.49×10^−14^	2.59×10^−11^
GO:0010033	Response to organic substance	Up	3.12×10^−14^	5.43×10^−11^
GO:0002521	Leukocyte differentiation	Up	4.00×10^−14^	6.96×10^−11^
GO:0002252	Immune effector process	Up	6.25×10^−14^	1.09×10^−10^
GO:0045619	Regulation of lymphocyte differentiation	Up	7.29×10^−14^	1.27×10^−10^
GO:0045058	T cell selection	Up	1.03×10^−13^	1.79×10^−10^
GO:0042127	Regulation of cell proliferation	Up	1.56×10^−13^	2.71×10^−10^
GO:0007610	Behavior	Up	3.16×10^−13^	5.51×10^−10^
GO:0009617	Response to bacterium	Up	9.56×10^−13^	1.66×10^−9^
GO:0001819	Positive regulation of cytokine production	Up	1.36×10^−12^	2.36×10^−9^
GO:0034097	Response to cytokine stimulus	Up	2.55×10^−12^	4.44×10^−9^
GO:0002253	Activation of immune response	Up	2.73×10^−12^	4.76×10^−9^
GO:0050670	Regulation of lymphocyte proliferation	Up	5.42×10^−12^	9.44×10^−9^
GO:0045061	Thymic T cell selection	Up	5.98×10^−12^	1.04×10^−8^
GO:0050900	Leukocyte migration	Up	6.39×10^−12^	1.11×10^−8^
GO:0070663	Regulation of leukocyte proliferation	Up	6.50×10^−12^	1.13×10^−8^
GO:0032944	Regulation of mononuclear cell proliferation	Up	6.50×10^−12^	1.13×10^−8^
GO:0030097	Hemopoiesis	Up	7.70×10^−12^	1.34×10^−8^
GO:0002443	Leukocyte mediated immunity	Up	9.29×10^−12^	1.62×10^−8^
GO:0045087	Innate immune response	Up	1.23×10^−11^	2.15×10^−8^
GO:0007243	Protein kinase cascade	Up	1.71×10^−11^	2.99×10^−8^
GO:0042102	Positive regulation of T cell proliferation	Up	2.24×10^−11^	3.90×10^−8^
GO:0002460	Adaptive immune response based on somatic recombination of immune receptors built from immunoglobulin superfamily domains	Up	2.59×10^−11^	4.51×10^−8^
GO:0002250	Adaptive immune response	Up	2.59×10^−11^	4.51×10^−8^
GO:0045597	Positive regulation of cell differentiation	Up	2.99×10^−11^	5.20×10^−8^
GO:0016477	Cell migration	Up	3.00×10^−11^	5.22×10^−8^
GO:0002757	Immune response-activating signal transduction	Up	3.64×10^−11^	6.34×10^−8^
GO:0010647	Positive regulation of cell communication	Up	3.66×10^−11^	6.38×10^−8^
GO:0008284	Positive regulation of cell proliferation	Up	4.73×10^−11^	8.23×10^−8^
GO:0048534	Hemopoietic or lymphoid organ development	Up	5.60×10^−11^	9.76×10^−8^
GO:0050671	Positive regulation of lymphocyte proliferation	Up	7.41×10^−11^	1.29×10^−7^
GO:0070665	Positive regulation of leukocyte proliferation	Up	9.30×10^−11^	1.62×10^−7^
GO:0032946	Positive regulation of mononuclear cell proliferation	Up	9.30×10^−11^	1.62×10^−7^
GO:0002764	Immune response-regulating signal transduction	Up	9.30×10^−11^	1.62×10^−7^
GO:0002449	Lymphocyte mediated immunity	Up	1.02×10^−10^	1.78×10^−7^
GO:0007242	Intracellular signaling cascade	Up	1.11×10^−10^	1.94×10^−7^
GO:0002237	Response to molecule of bacterial origin	Up	1.23×10^−10^	2.14×10^−7^
GO:0045089	Positive regulation of innate immune response	Up	1.61×10^−10^	2.80×10^−7^
GO:0045621	Positive regulation of lymphocyte differentiation	Up	1.62×10^−10^	2.82×10^−7^
GO:0031349	Positive regulation of defense response	Up	1.78×10^−10^	3.10×10^−7^
GO:0002520	Immune system development	Up	1.86×10^−10^	3.25×10^−7^
GO:0048870	Cell motility	Up	2.74×10^−10^	4.76×10^−7^
GO:0051674	Localization of cell	Up	2.74×10^−10^	4.76×10^−7^
GO:0009615	Response to virus	Up	3.09×10^−10^	5.39×10^−7^
GO:0042129	Regulation of T cell proliferation	Up	3.30×10^−10^	5.74×10^−7^
GO:0032496	Response to lipopolysaccharide	Up	3.58×10^−10^	6.24×10^−7^
GO:0007159	Leukocyte adhesion	Up	4.19×10^−10^	7.29×10^−7^
GO:0045580	Regulation of T cell differentiation	Up	5.34×10^−10^	9.31×10^−7^
GO:0043065	Positive regulation of apoptosis	Up	5.62×10^−10^	9.78×10^−7^
GO:0043068	Positive regulation of programmed cell death	Up	6.60×10^−10^	1.15×10^−6^
GO:0009967	Positive regulation of signal transduction	Up	6.94×10^−10^	1.21×10^−6^
GO:0010942	Positive regulation of cell death	Up	7.34×10^−10^	1.28×10^−6^
GO:0051092	Positive regulation of NF-κB transcription factor activity	Up	9.07×10^−10^	1.58×10^−6^
GO:0019882	Antigen processing and presentation	Up	9.44×10^−10^	1.64×10^−6^
GO:0045088	Regulation of innate immune response	Up	1.03×10^−9^	1.79×10^−6^
GO:0019221	Cytokine-mediated signaling pathway	Up	1.44×10^−9^	2.52×10^−6^
GO:0019724	B cell mediated immunity	Up	1.55×10^−9^	2.71×10^−6^
GO:0002504	Antigen processing and presentation of peptide or polysaccharide antigen via MHC class II	Up	2.18×10^−9^	3.79×10^−6^
GO:0002221	Pattern recognition receptor signaling pathway	Up	5.25×10^−9^	9.14×10^−6^
GO:0032844	Regulation of homeostatic process	Up	5.62×10^−9^	9.78×10^−6^
GO:0008219	Cell death	Up	8.22×10^−9^	1.43×10^−5^
GO:0032101	Regulation of response to external stimulus	Up	8.42×10^−9^	1.47×10^−5^
GO:0033077	T cell differentiation in the thymus	Up	8.44×10^−9^	1.47×10^−5^
GO:0006874	Cellular calcium ion homeostasis	Up	9.61×10^−9^	1.67×10^−5^
GO:0016265	Death	Up	9.82×10^−9^	1.71×10^−5^
GO:0002683	Negative regulation of immune system process	Up	1.09×10^−8^	1.90×10^−5^
GO:0060326	Cell chemotaxis	Up	1.10×10^−8^	1.91×10^−5^
GO:0002758	Innate immune response-activating signal transduction	Up	1.32×10^−8^	2.30×10^−5^
GO:0002218	Activation of innate immune response	Up	1.32×10^−8^	2.30×10^−5^
GO:0051090	Regulation of transcription factor activity	Up	1.43×10^−8^	2.49×10^−5^
GO:0055074	Calcium ion homeostasis	Up	1.44×10^−8^	2.51×10^−5^
GO:0008283	Cell proliferation	Up	1.52×10^−8^	2.65×10^−5^
GO:0016064	Immunoglobulin mediated immune response	Up	1.60×10^−8^	2.79×10^−5^
GO:0006915	Apoptosis	Up	1.80×10^−8^	3.14×10^−5^
GO:0048545	Response to steroid hormone stimulus	Up	1.98×10^−8^	3.44×10^−5^
GO:0012501	Programmed cell death	Up	2.53×10^−8^	4.41×10^−5^
GO:0006875	Cellular metal ion homeostasis	Up	2.69×10^−8^	4.68×10^−5^
GO:0042035	Regulation of cytokine biosynthetic process	Up	3.36×10^−8^	5.85×10^−5^
GO:0045582	Positive regulation of T cell differentiation	Up	3.73×10^−8^	6.49×10^−5^
GO:0051091	Positive regulation of transcription factor activity	Up	4.64×10^−8^	8.07×10^−5^
GO:0001816	Cytokine production	Up	5.16×10^−8^	8.98×10^−5^
GO:0055065	Metal ion homeostasis	Up	5.22×10^−8^	9.09×10^−5^
GO:0042325	Regulation of phosphorylation	Up	5.84×10^−8^	1.02×10^−4^
GO:0045060	Negative thymic T cell selection	Up	6.40×10^−8^	1.11×10^−4^
GO:0040017	Positive regulation of locomotion	Up	7.34×10^−8^	1.28×10^−4^
GO:0007249	I-κB kinase/NF-κB cascade	Up	7.52×10^−8^	1.31×10^−4^
GO:0042108	Positive regulation of cytokine biosynthetic process	Up	7.63×10^−8^	1.33×10^−4^
GO:0006917	Induction of apoptosis	Up	8.00×10^−8^	1.39×10^−4^
GO:0012502	Induction of programmed cell death	Up	8.50×10^−8^	1.48×10^−4^
GO:0051101	Regulation of DNA binding	Up	1.01×10^−7^	1.75×10^−4^
GO:0002697	Regulation of immune effector process	Up	1.03×10^−7^	1.80×10^−4^
GO:0030595	Leukocyte chemotaxis	Up	1.27×10^−7^	2.22×10^−4^
GO:0044093	Positive regulation of molecular function	Up	1.28×10^−7^	2.22×10^−4^
GO:0051174	Regulation of phosphorus metabolic process	Up	1.29×10^−7^	2.24×10^−4^
GO:0019220	Regulation of phosphate metabolic process	Up	1.29×10^−7^	2.24×10^−4^
GO:0050864	Regulation of B cell activation	Up	1.33×10^−7^	2.31×10^−4^
GO:0043383	Negative T cell selection	Up	1.42×10^−7^	2.47×10^−4^
GO:0050730	Regulation of peptidyl-tyrosine phosphorylation	Up	1.59×10^−7^	2.77×10^−4^
GO:0007155	Cell adhesion	Up	1.65×10^−7^	2.87×10^−4^
GO:0022610	Biological adhesion	Up	1.70×10^−7^	2.97×10^−4^
GO:0043388	Positive regulation of DNA binding	Up	2.11×10^−7^	3.68×10^−4^
GO:0030005	Cellular di-, tri-valent inorganic cation homeostasis	Up	2.29×10^−7^	3.99×10^−4^
GO:0002822	Regulation of adaptive immune response based on somatic recombination of immune receptors built from immunoglobulin superfamily domains	Up	2.62×10^−7^	4.55×10^−4^
GO:0007204	Elevation of cytosolic calcium ion concentration	Up	2.67×10^−7^	4.65×10^−4^
GO:0002819	Regulation of adaptive immune response	Up	3.07×10^−7^	5.35×10^−4^
GO:0006916	Anti-apoptosis	Up	3.21×10^−7^	5.58×10^−4^
GO:0055066	Di-, tri-valent inorganic cation homeostasis	Up	4.78×10^−7^	8.33×10^−4^
GO:0051480	Cytosolic calcium ion homeostasis	Up	5.76×10^−7^	1.00×10^−3^
GO:0006468	Protein amino acid phosphorylation	Up	5.91×10^−7^	1.03×10^−3^
GO:0051099	Positive regulation of binding	Up	5.97×10^−7^	1.04×10^−3^
GO:0042592	Homeostatic process	Up	7.89×10^−7^	1.37×10^−3^
GO:0050871	Positive regulation of B cell activation	Up	9.20×10^−7^	1.60×10^−3^
GO:0051050	Positive regulation of transport	Up	9.32×10^−7^	1.62×10^−3^
GO:0001932	Regulation of protein amino acid phosphorylation	Up	1.09×10^−6^	1.90×10^−3^
GO:0030003	Cellular cation homeostasis	Up	1.11×10^−6^	1.94×10^−3^
GO:0045086	Positive regulation of interleukin-2 biosynthetic process	Up	1.37×10^−6^	2.39×10^−3^
GO:0051098	Regulation of binding	Up	1.54×10^−6^	2.68×10^−3^
GO:0051047	Positive regulation of secretion	Up	1.86×10^−6^	3.23×10^−3^
GO:0002224	Toll-like receptor signaling pathway	Up	2.11×10^−6^	3.68×10^−3^
GO:0045637	Regulation of myeloid cell differentiation	Up	2.16×10^−6^	3.76×10^−3^
GO:0045059	Positive thymic T cell selection	Up	2.46×10^−6^	4.29×10^−3^
GO:0002429	Immune response-activating cell surface receptor signaling pathway	Up	3.05×10^−6^	5.30×10^−3^
GO:0009725	Response to hormone stimulus	Up	3.08×10^−6^	5.37×10^−3^
GO:0051241	Negative regulation of multicellular organismal process	Up	3.36×10^−6^	5.85×10^−3^
GO:0040012	Regulation of locomotion	Up	3.76×10^−6^	6.55×10^−3^
GO:0070482	Response to oxygen levels	Up	3.85×10^−6^	6.71×10^−3^
GO:0051270	Regulation of cell motion	Up	4.00×10^−6^	6.96×10^−3^
GO:0010740	Positive regulation of protein kinase cascade	Up	4.10×10^−6^	7.15×10^−3^
GO:0006793	Phosphorus metabolic process	Up	4.14×10^−6^	7.20×10^−3^
GO:0006796	Phosphate metabolic process	Up	4.14×10^−6^	7.20×10^−3^
GO:0045577	Regulation of B cell differentiation	Up	4.48×10^−6^	7.80×10^−3^
GO:0002495	Antigen processing and presentation of peptide antigen via MHC class II	Up	4.86×10^−6^	8.47×10^−3^
GO:0019886	Antigen processing and presentation of exogenous peptide antigen via MHC class II	Up	4.86×10^−6^	8.47×10^−3^
GO:0051272	Positive regulation of cell motion	Up	4.98×10^−6^	8.67×10^−3^
GO:0002768	Immune response-regulating cell surface receptor signaling pathway	Up	5.12×10^−6^	8.92×10^−3^
GO:0055080	Cation homeostasis	Up	5.58×10^−6^	9.72×10^−3^
GO:0044057	Regulation of system process	Down	1.01×10^−13^	1.67×10^−10^
GO:0009719	Response to endogenous stimulus	Down	1.25×10^−12^	2.07×10^−9^
GO:0009725	Response to hormone stimulus	Down	2.19×10^−11^	3.62×10^−8^
GO:0007267	Cell-cell signaling	Down	2.88×10^−11^	4.77×10^−8^
GO:0010033	Response to organic substance	Down	1.52×10^−10^	2.52×10^−7^
GO:0007166	Cell surface receptor linked signal transduction	Down	3.97×10^−10^	6.57×10^−7^
GO:0048878	Chemical homeostasis	Down	6.67×10^−10^	1.10×10^−6^
GO:0050678	Regulation of epithelial cell proliferation	Down	8.15×10^−10^	1.35×10^−6^
GO:0042127	Regulation of cell proliferation	Down	8.51×10^−10^	1.41×10^−6^
GO:0050679	Positive regulation of epithelial cell proliferation	Down	7.87×10^−9^	1.30×10^−5^
GO:0007610	Behavior	Down	8.57×10^−9^	1.42×10^−5^
GO:0042592	Homeostatic process	Down	1.18×10^−8^	1.95×10^−5^
GO:0050801	Ion homeostasis	Down	6.17×10^−8^	1.02×10^−4^
GO:0055065	Metal ion homeostasis	Down	9.62×10^−8^	1.59×10^−4^
GO:0006873	Cellular ion homeostasis	Down	1.39×10^−7^	2.30×10^−4^
GO:0055082	Cellular chemical homeostasis	Down	1.69×10^−7^	2.80×10^−4^
GO:0032870	Cellular response to hormone stimulus	Down	2.14×10^−7^	3.54×10^−4^
GO:0007169	Transmembrane receptor protein tyrosine kinase signaling pathway	Down	2.36×10^−7^	3.91×10^−4^
GO:0019725	Cellular homeostasis	Down	3.35×10^−7^	5.53×10^−4^
GO:0007167	Enzyme linked receptor protein signaling pathway	Down	3.42×10^−7^	5.65×10^−4^
GO:0008284	Positive regulation of cell proliferation	Down	4.78×10^−7^	7.91×10^−4^
GO:0040012	Regulation of locomotion	Down	5.05×10^−7^	8.35×10^−4^
GO:0006875	Cellular metal ion homeostasis	Down	6.10×10^−7^	1.01×10^−3^
GO:0008016	Regulation of heart contraction	Down	6.95×10^−7^	1.15×10^−3^
GO:0048511	Rhythmic process	Down	1.92×10^−6^	3.17×10^−3^
GO:0055080	Cation homeostasis	Down	2.63×10^−6^	4.36×10^−3^
GO:0019932	Second-messenger-mediated signaling	Down	3.16×10^−6^	5.23×10^−3^
GO:0040017	Positive regulation of locomotion	Down	3.58×10^−6^	5.92×10^−3^
GO:0055074	Calcium ion homeostasis	Down	3.90×10^−6^	6.45×10^−3^
GO:0010863	Positive regulation of phospholipase C activity	Down	4.14×10^−6^	6.84×10^−3^
GO:0007202	Activation of phospholipase C activity	Down	4.14×10^−6^	6.84×10^−3^
GO:0007242	Intracellular signaling cascade	Down	4.31×10^−6^	7.14×10^−3^
GO:0051969	Regulation of transmission of nerve impulse	Down	5.40×10^−6^	8.93×10^−3^
GO:0010518	Positive regulation of phospholipase activity	Down	5.88×10^−6^	9.73×10^−3^

GO, gene ontology; FDR, false discovery rate; NF, nuclear factor; MHC, major histocompatibility complex.
